# Feasibility and Acceptability of a Cognitive Behavioral Therapy-Based Smartphone App for Smoking Cessation in China: A Single-Group Cohort Study

**DOI:** 10.3389/fpsyt.2021.759896

**Published:** 2022-03-03

**Authors:** Yanhui Liao, Jinsong Tang

**Affiliations:** ^1^Department of Psychiatry, Sir Run Run Shaw Hospital, Zhejiang University School of Medicine, Hangzhou, China; ^2^Key Laboratory of Medical Neurobiology of Zhejiang Province, Hangzhou, China

**Keywords:** feasibility, acceptability, smartphone app, smoking cessation, cognitive behavioral therapy (CBT), China

## Abstract

**Background:**

Previous research has suggested that mobile phone applications (apps) may potentially increase quit rates. The purpose of this single-group cohort study sought to examine the feasibility and acceptability of a novel smartphone-based smoking cessation app designed for smoking cessation in China: smoking quit rate.

**Methods:**

A total of 180 smokers from two cities of mainland China with willingness to make a quit attempt were invited to this smoking cessation app program, a cognitive behavioral theory (CBT)-based smoking cessation intervention via a smartphone app. Participants received 37- to 44-day intervention (including 7- to 14-day pre-quit preparation and 33-day intervention from quit date). Feasibility and acceptability of the program, and smoking status were assessed at baseline stage (initial installation), pre-quit stage, and post-quit stage (days 7, 15, and 33 after quit date).

**Results:**

A total of 163 (90.6%) participants completed the study. Among them, 76–89% of the participants logged into the app ≥1 time per day across stages (at baseline, during pre-quit stage, and on days 7, 15, and 33 of post-quit stage); approximately 90% of the participants were satisfied with the app across stages. A significant rise in self-reported overall satisfaction with the app is observed from baseline (93% at Time 1) to the end of the program (98% at Time 2, 33 days after quit date) (*p* = 0.021). Participants who believed/agreed this app can help them to quit smoking significantly increased from 69% at baseline to 97% at day 33 after quit date (*p* < 0.001). Participants were satisfied with most (80–90%) of the features, especially the information feature. Intention-to-treat analysis showed that the percentage of 33-day self-reported continuous prevalence abstinence was 63.9%, and 7-day point prevalence abstinence rate was 81.7, 87.2, and 77.8% on days 7, 15, and 33 after quit date, respectively.

**Conclusions:**

This study demonstrated the feasibility and acceptability of the smartphone app intervention for smoking cessation and introduced a new digital treatment model, which is expected to overcome barriers facing accessing traditional in-person smoking cessation services and extend nationwide smoking cessation services in China.

## Introduction

Tobacco use (mainly cigarette smoking) is one of the most serious but avoidable public health problems in the world, globally causing 7.69 million of premature deaths and 200 million disability-adjusted life-years in a single year, 2019 (1). With more than 300 million current smokers, China now consumes about 40% of the total cigarettes in the world and experiences a large increase in consumption in urban men over the past three decades (2). In 2006, China ratified the Framework Convention on Tobacco Control (FCTC) of the WHO and implemented strong tobacco control strategies. China has made progress on tobacco control over the last decade or two. However, the prevalence of current smoking remains extremely high; still, more than half of adult men are current smokers in China (2).

The Chinese government released the Healthy China (HC) 2030 blueprint in October 2016 as a national strategy with sets of targets to promote health at a national level (3). One of the targets in the HC 2030 strategy sets is to reduce prevalence of smoking from 27.7% in 2015 to 20% by 2030 (4). A potential key step to achieve the targeted reduction in smoking by 2030 is to provide nationwide smoking cessation services. Since the first cessation clinic in China was set up in 1996, China now has more than 800 cessation clinics, but only a few patients (one to two patients/week/hospital) are willing to seek assistance in these smoking cessation clinics (5). A study showed that among those who experienced smoking relapse, more than 90% of them did not receive any treatment or counseling on smoking cessation (2). In China, the main reasons why current interventions are not commonly used are smoking prevalence of male physicians is relatively high, while the quit rate is relatively low, and standard smoking cessation practices are rarely provided by health service providers (HSPs) (6–8); these are the main reasons why current interventions are not commonly used in China. Thus, a few of the smokers are able to successfully quit smoking, and even many of them have intentions (9, 10).

Mobile phone-based smoking cessation services may provide a new strategy to meet the gap between the demanding need for those treatment-seeking smokers and the lack of evidence-based, widely accessible, and feasible smoking cessation services. Smoking cessation services delivered via mobile phone have a number of advantages. First, these digital health services are relatively inexpensive or even free. Second, once developed, interventions delivered via mobile phone can be widely accessible. Additionally, participants can receive these services anonymously. In response to these advantages, mobile phone-based smoking cessation services to help quit smoking have been widely available in many countries. The evidence on the effect of smoking cessation interventions delivered by mobile phones to people who want to quit smoking was well-documented across countries (11). Our previous randomized clinical trial (RCT) of “Happy Quit” (a text messaging-based smoking cessation intervention) showed its efficacy in quitting smoking in China (12). Mobile phone text messaging and app-based smoking cessation interventions are the most popular ones delivered remotely. Compared with text messaging, the smartphone apps are potentially more flexible and cost effective given the fact that the majority of China's adults own smartphones, and smartphone apps for smoking cessation are increasingly used in China to help smokers quit. However, the content analysis of the apps found that these apps have very low level of adherence to standard clinical practice guidelines (13). Up to the present, there are no assessment reports for the feasibility and acceptability of smartphone apps for smoking cessation in China. Furthermore, there is no evidence-based smoking cessation app in China.

In response to this, a smoking cessation app was developed following the clinical practice guidelines to help Chinese smokers who want to quit. Given that cognitive behavioral approaches were the most promising interventions for smoking cessation (14), the content in the app mainly was based on cognitive behavioral theory (CBT). A broad range of issues of quitting smoking can be addressed by CBT, from self-efficacy and motivation to relapse prevention (such as stress management, concerns about weight gaining), augmenting maintenance of abstinence in treatment-seeking individuals. More detailed context in the app and app features were described in our published protocol (15).

The objectives of this study were evaluating the feasibility and acceptability (preliminary by measuring acceptance and satisfaction toward the app of users), and the potential efficacy (preliminary by evaluating self-reported 7-day point quit rate at weeks 1, 2, and 4 as well as 33-day continuous abstinence rate) of this Chinese CBT-based smoking cessation app. Given that CBT (16, 17) is the current standard in behavioral intervention for smoking cessation, we postulate that this CBT-based app would be highly feasible and acceptable in China. Considering the efficacy of CBT-based smoking cessation app (self-reported 7-day point quit rate was 44.5% in the “Quit Genius” intervention group) in the United Kingdom (18), we also tested the hypotheses that participation in this intervention would lead to a significant improvement of at least 40% in self-reported 7-day point quit rate as well as 33-day continuous abstinence rate.

## Methods

### Study Design

This was an internet-based, open-label, single-group study of a Chinese CBT-based app program. With 7–14 days of pre-quitting intervention and 33 days of post-quitting intervention, this study aimed to evaluate the feasibility and acceptability of the Chinese CBT-based mobile app program by measuring levels of aesthetics, functionality, engagement, information quality of the app (each question used a five-point Likert scale: “strongly agree,” “somewhat agree,” “neither agree nor disagree,” “somewhat disagree,” and “strongly disagree”), and satisfaction with the app; analyze changes in attitude toward quitting smoking from baseline (Time 1) to the end of the program (Time 2); and evaluate the improvement in self-reported 7-day point quit rate at days 7, 15, and 2-week point quit rate at day 33 after quit date.

### Recruitment and Study Participants

**Figure 1:**
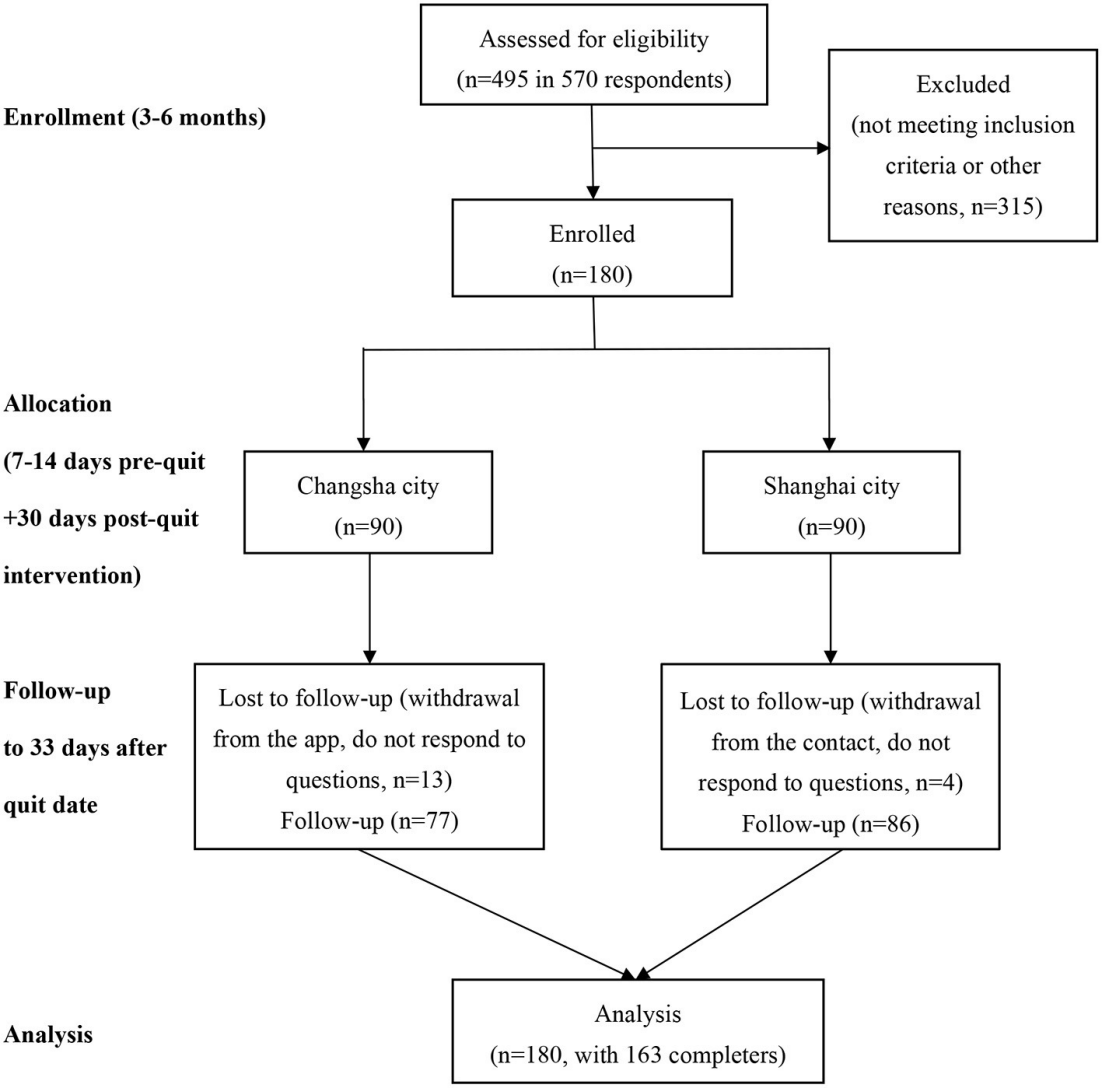
Flowchart for the study. Note for exclusion: “not meeting inclusion criteria” including had no or little motivation to quit, aged more than 45 years, smoked less than five cigarettes per day; “other reasons” including had no more interest in this study any more, provided incorrect contact number.

From middle April to middle May 2020, Participants from Changsha City or Shanghai City in mainland China were recruited via web-based advertisements and social media (WeChat, QQ, and WeiBo). Changsha, a typical, relatively slow-paced city, is the capital city of Hunan Province in the central south China. Shanghai is the biggest fast-paced city and a financial hub in the central coast of China. Among 570 respondents, a total of 495 participants were recruited through online screener and telephone interview to determine eligibility. Of the screened, 315 did not meet preliminary inclusion/exclusion criteria, including having no or little motivation to quit, more than 45 years old, and smoking less than five cigarettes per day. Participants who completed the first-round screening were invited to complete a pre-intervention questionnaire. A total of 180 participants (90 participants from Changsha City and 90 participants from Shanghai City) were included. Among them, 163 participants (90.6%) completed the study (77 participants from Changsha and 86 participants from Shanghai). Figure 1 shows a CONSORT flowchart for the study. The eligibility criteria for participants were as follows: being aged 20–45 years, being able to speak and read simplified Chinese, current cigarettes smokers who smoke five or more cigarettes per day and have at least 1-year smoking experience, willing to use the app to assist smoking cessation, have moderate and high motivation for smoking cessation [defined as those who rate >4 at the single Motivation for Terminating Smoking Scale (MTSS), levels of motivation are 1: “I don't want to stop smoking”; 2: “I think I should stop smoking but don't really want to”; 3: “I want to stop smoking but haven't thought about when”; 4: “I REALLY want to stop smoking but I don't know when I will”; 5: “I want to stop smoking and hope soon”; 6: “I REALLY want to stop smoking and intend to in the next 3 months”; 7: “I REALLY want to stop smoking and intend to in the next month”] (19); and iOS phone users (iPhone 6 and above versions). Participants who had already started to quit or using any smoking cessation treatment at the time of registration or participants currently experiencing severe mental illness were excluded in this study. This trial was registered at clinicaltrials.gov (trial registration number: NCT04421170).

### “Cognitive Behavioral Theory-Based Smoking Cessation” App Software

The cigarette smoking cessation app software was developed based on the principles of CBT and the clinical experience, scientific or empirical evidence of smoking cessation, and was supervised by Dr. Yanhui Liao and Dr. Jinsong Tang. More details about the CBT embedding smoking cessation program were described in the protocol (15). Representation of screenshots of the app are shown in Supplementary File 1, including the features of the app register and login page, app main page of preparation stage, an example of CBT training task content—breathing relaxation training, smoking record input page, quit manifesto page, main page of post-cessation stage, an example of CBT training task content—periodic self-reward, an example of notification, SOS (emergency help button) function page, quitting benefits tracker page, and Twitter-like forum and information module (see Supplementary Figures 1–11).

### Procedure

An on-site appointment letter was sent to each eligible participant (*n* = 180). During the on-site appointment, a research assistant explained the purpose, all procedures and measurements, and potential risks and benefits of the study to each participant. All participants provided informed consent, received instructions to download the app on their mobile phone and to use it, and completed a baseline feedback questionnaire about their experience of download and register process and the first impression on the app design (including overall satisfaction and easiness of registration, etc.). Participants were required to select a quit date between days 7 and 14 and chose the way of quitting (either gradual or abrupt smoking cessation) in the app. During the 7–14 days of pre-quitting intervention period, the participants were asked to engage with the “CBT-based smoking cessation” app on a daily basis and report how satisfied they are with the following features of the app: aesthetics, functionality, engagement, and information. During the 33 days of post-quitting intervention period, participants were required to report their smoking status on days 7, 15, and 33 by a web-based questionnaire (within the app, each smoking attempt was encouraged to be recorded as well).

### Definition

#### Overall Satisfaction

Satisfaction with app's features of aesthetics, functionality, engagement, and information.

#### Quitter

A participant who smoked no more than five cigarettes from quit day to day 33 (i.e., self-reported 33-day continuous abstinence).

#### Non-quitter

A participant who smoked more than five cigarettes from quit day to day 33.

#### Perceived Effect on Smoking Cessation

A participant who believed or agreed this CBT-based smoking cessation app can help him/her to successfully quit smoking.

#### Severity of Nicotine Dependence

Severity of nicotine dependence was measured by Fagerström Test for Cigarette Dependence (FTCD). The total FTCD score ranged from 0 to 10, a score ≤6 indicates light to moderate dependence, a score ≥7 indicates heavy dependence.

### Measures and Outcomes

#### Demographic and Smoking Characteristics

Demographics include age, gender, education, monthly household income, marital status; smoking characteristics include years of smoking, smoked cigarettes per day, quit history, motivation and preferred methods for quitting (gradually or abruptly), smoking craving, and severity of nicotine dependence assessed by FTCD.

#### Satisfaction With “Cognitive Behavioral Theory-Based Smoking Cessation” App

Participants were asked to engage with the app on a daily basis and report the overall satisfaction with the app and how satisfied they were with the features of the app in terms of aesthetics, functionality, engagement, and information quality. Each question used a five-point Likert scale: from “strongly agree,” “somewhat agree,” to “neither agree nor disagree,” “somewhat disagree,” and “strongly disagree.” The percentage of satisfaction with the app was calculated by the percentage of “strongly agree” and “somewhat agree” response among the response of all the participants; overall satisfaction across stages [from baseline (Time 1) to the end of the program (Time 2)] was measured by a five-point Likert scale: from “very satisfied,” “satisfied,” to “can't tell,” “unsatisfied,” and “very unsatisfied”; perceived effect on smoking cessation across stages was measured by a five-point Likert scale: from “strongly believe/agree,” “believe/agree,” to “not sure,” “unbelieve/disagree,” and “strongly unbelieve/disagree.” Recommendation to friends was assessed by asking the question on “How would you likely to recommend this app to friends,” measured from extremely unlikely (0) to extremely likely (10).

#### Self-Reported Smoking Abstinence

Self-reported 7-day point abstinence on days 7 and 15, 2-week point abstinence on day 33, and continues abstinence on day 33 after the quit date of each participant was collected during the trial. It was defined as smoked no more than five cigarettes in the past 7 or 33 days since quitting, measured via response to the following items: (a) “How many cigarettes have you smoked in the past week” on days 7 and 15 after quit data and (b) “How many cigarettes have you smoked in the past 2 weeks on day 33 after quit data.” The added-up responses were further categorized into two groups “no more than 5” or “more than 5”.

We tested the hypotheses that more than half of the participants in this program would be satisfied with the features of the app, and participation in this intervention would lead to significant improvement of short-term quit rate (at least 40% in self-reported 33-day continuous abstinence). Additionally, we postulated that this CBT-based app would be highly feasible and acceptable in China.

### Safety

Safety was monitored and assessed by reviewing the collection, evaluation, and analysis of subject-reported spontaneous adverse events (AEs). No participants reported serious adverse events in this study.

### Data Collection

Most data (including demographic and smoking characteristics, and users' feedback to app) were collected online by WenJuanXing, a Chinese online market research website that provides professional online questionnaire survey or data collection for clinical trials (20). A user-specified file (Microsoft Excel version) was downloaded from the database website. The app is mainly providing smoking cessation interventions, and only data of average login times (number of times opened the app) across all stages were collected from it. All data were monitored by How-To NPD consulting company in which personal data were de-identified to ensure the privacy of the subject.

### Statistical Analyses

All analyses were done on the intention-to-treat (ITT) principle (21). Participants who failed to respond to the follow-up endpoint were retained in the analyses and classified as smokers. Given the electronic nature of the data collection and only a short-term intervention, only 17 (9.44%) participants did not complete the study. When all data have been obtained, they were analyzed by R programming. Descriptive statistics were applied for demographic and smoking characteristics at baseline; ANOVA (for continuous variables) and χ^2^ test (for categorical variables) were applied to compare the demographic information smoking behavior at baseline between groups of participants from Changsha City and Shanghai City. All tests were two tailed. A two-sided *p* < 0.05 was applied to determine statistical significance.

## Results

### Participant Characteristics

Demographic and smoking characteristics in all baseline participants (*n* = 180) and between participants from Changsha City (*n* = 90) and Shanghai City (*n* = 90) are shown in Table 1.

**Table 1:** Demographic and smoking characteristics in all samples, between participants from Changsha City and Shanghai City.

	**All samples (*N* = 180)**	**Changsha (*N* = 90)**	**Shanghai (*N* = 90)**	***p*-value**
**Demographic characteristics**				
Age (mean ± SD)	31.6 ± 6.33	30.6 ± 5.81	32.5 ± 6.71	0.046
20–30 years old (*n*, %)	85, 47.2%	49, 54.4%	36, 40%	0.07
31–45 years old (*n*, %)	95, 52.7%	41, 45.6%	54, 60%	
**Gender (** ***n*** **, %)**				
Male	161, 89.4%	80, 88.9%	81, 90%	1
Female	19, 10.5%	10, 11.1%	9, 10%	
**Education (n, %)**				
High school to college	93, 51.7%	57, 63.3%	36, 40%	0.003
Bachelor and above	87, 48.3%	33, 36.7%	54, 60%	
**Monthly household income (** ***n*** **, %)**				
<15,000 CNY	58, 32.2%	45, 50%	13, 14.4%	<0.001
≥15,000 CNY	122, 67.8%	45, 50%	77, 85.5%	
**Marital status (** ***n*** **, %)**				
Unmarried	74, 41.1%	36, 40%	38, 42.2%	0.899
Married (without child/children)	17, 9.4%	8, 8.9%	9,10%	
Married (with child/children)	89, 49.4%	46, 51.1%	43, 47.8%	
**Smoking characteristics**				
**Years of smoking (** ***n*** **, %)**				
1–5 years	67, 37.2%	38, 42.2%	29, 32.2%	0.217
>5 years	113, 62.8%	52, 57.8%	61, 67.8%	
**Smoked cigarettes per day (** ***n*** **, %)**				
5–10 cigarettes	80, 44.4%	39, 43.3%	41, 45.6%	0.881
>10 cigarettes	100, 55.6%	51, 56.7%	49, 54.4%	
FTCD mean score (mean ± SD)	4.8 ± 2.03	4.1 ± 2.18	5.4 ± 1.66	<0.001
Light to moderate smoking (*n*, %)	87, 48.3%	58, 64.4%	29, 32.2%	<0.001
Heavy smoking (*n*, %)	93, 51.7%	32, 35.6%	61, 67.8%	
**Quit attempts during the past 12 months (** ***n*** **, %)**				
No	0, 0	0, 0	0, 0	1
Yes	180, 100%	90, 100%	90, 100%	
**Selection of quitting smoking way^#^** **(*****n*****, %)**				
Gradually quit	111, 68.1%	48, 62.3%	63, 73.3%	0.185
Abruptly quit	52, 31.9%	29, 37.7%	23, 26.7%	
**Days of preparation stage^#^** **(*****n*****, %)**				
7 days	93, 57.1%	58, 75.3%	35, 40.7%	<0.001
8–14 days	70, 42.9%	19, 24.7%	51, 59.3%	

*FTCD, the Fagerström Test for Cigarette Dependence; light to moderate smoking: total FTCD mean scores ≤6; heavy smoking: total FTCD mean scores between 7 and 10*.

# *Data from app backstage (N = 163)*.

### Satisfaction With “Cognitive Behavioral Theory-Based Smoking Cessation” App

More than 70% (76–89%) of the participants logged into the app more than one time per day. Average login times across all stages are shown in Supplementary Table 4. Login time by quitters and non-quitters, and by Shanghai City and Changsha City during the post-quit stage (%) are shown in Supplementary Figure 12. Compared with the initial stage, participants are more likely to recommend to friends at the end of the program (Supplementary Figure 13).

**Figure 2:**
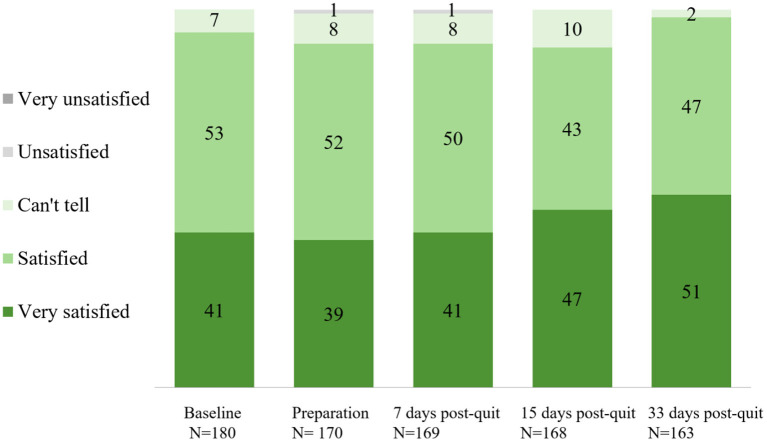
Overall satisfaction with the cognitive behavioral theory (CBT)-based smoking cessation app across stages (%).

**Figure 3:**
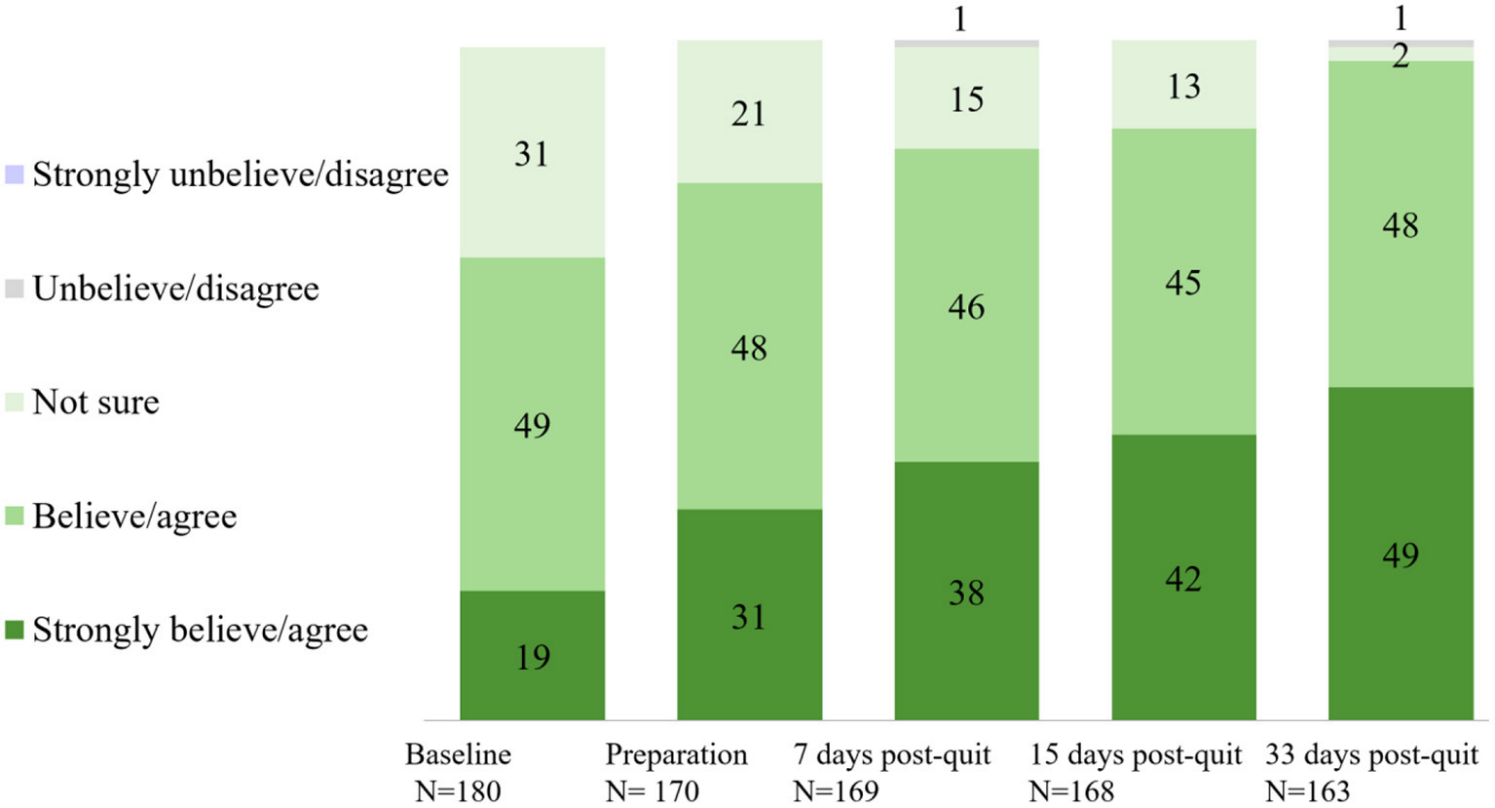
Participants who believed/agreed this app can help them to quit smoking (%).

Overall satisfaction with the app across stages is shown in Figure 2. Approximately 90% of the participants were satisfied with the app across stages. A significant rise in self-reported overall satisfaction with the app was observed from baseline (93% at Time 1) to the end of the program (98% at Time 2, day 33 after quit date) (*p* = 0.021). Perceived effect on smoking cessation (believed/agreed this app can help them to quit smoking) across stages is shown in Figure 3. Participants who believed/agreed this app can help them to quit smoking significantly increased from 69% at baseline to 97% at day 33 after quit date (*p* < 0.001). Overall satisfaction with the app and participants who believed/agreed this app can help them to quit smoking by subgroup at baseline, during pre-quit stage, and on days 7, 15, and 33 of the post-quit stage are shown in Supplementary Tables 1, 2, respectively.

Self-reported satisfaction with the four features (aesthetics, functionality, engagement, and information) of the app is shown in Table 2. Participants were satisfied with most (80% to more than 90%) of the features, especially the information feature.

**Table 2:** Percentage of participants who were satisfied with the app's features.

**Features**	**Satisfied/agreed (%)**
**Aesthetics**	
•The design and layout are attractive	86
•Color and design style are attractive	85
•The icon is easy to understand	85
•The layout is clear without any confusion	85
•The overall UI design	89
**Functionality**	
•Easy to find and download the app	77
•Easy to complete the registration	94
•This app is easy to navigate	87
•I would imagine that most people would learn to use this app very quickly	90
•This app is easy to navigate	89
•Information on the app is easy to find	88
•The app responds fast	91
•The app is stable	91
•The overall story line (dessert, ocean, and new chapter)	91
•This app is easy to navigate	95
•I know each function well	80
•The rules and instructions in this app are clear to me	91
•How easy to comply with the assigned tasks during the whole usage experience	93
**Engagement**	
•The survey is customized for me	78
•It is fun and motivating to use	85
•The task is interesting and exciting	68
•The task is interactive and motivating	76
•The task is relevant to me	83
•The assignments are customized for me	68
•The overall usage experience	90
•How would you describe your overall experience with the product?	98
•After these days' usage, how likely would you recommend this app to a friend who wants to quit smoking?	75
•Would you like to stay in the app to share the cessation experience to help others?	86
•Would you like to continue to use this app if possible?	95
**Information**	
•How much do you agree “this app will help me to quit smoking successfully”?	87
•The tasks are helpful on quitting smoking	91
•What would you say about the end result of this quit attempt with app assistant by now?	97
•How would you describe your craving of smoking during past 2 weeks?	99
•How would you describe your craving of smoking during the past 1 month?	98
•Do you think this app is effective in helping smoking cessation?	97

### Intervention Effects: Self-Reported Abstinence Rate

Based on the ITT principle, the percentage of 33-day self-reported continuous prevalence abstinence was 63.9% (*n* = 115). The self-reported 33-day continuous abstinence rate was higher in participants from Shanghai City (71%) than those from Changsha City (44%) (*p* < 0.001). Self-reported 7-day point prevalence smoking abstinence on day 7 after quit date was 81.7% (*n* = 147), on day 15 after quit date was 87.2% (*n* = 157), and 2-week point prevalence on day 33 after quit date was 77.8% (*n* = 140).

The differences in demographic and smoking characteristics between quitters (self-reported 33-day continuous abstinence) and non-quitters who completed the study (*n* = 163) are shown in Supplementary Table 3.

## Discussion

### Principal Findings

To the best of our knowledge, this single-group cohort study is the first one to assess the feasibility and acceptability of CBT-based smartphone app for treatment-seeking cigarette smokers in China, a country with more than 300 million current cigarette smokers. This study found that over 90% of participants completed the program, and rated program quality and satisfaction very high (90% of participants satisfied with the app across stages). Also, over 60% of the participants were able to stop smoking from the quit date to the end of the program (33 days after quit date). These findings demonstrated the feasibility, acceptability, and preliminary efficacy of the CBT-based smartphone app for smoking cessation in China.

### Program Satisfaction

This study found that the majority of participants were highly satisfied with the app across stages (from baseline to the end of the program) and across multiple app features, including aesthetics, functionality, engagement, and information. Participants were especially satisfied with the feature on information. The findings are similar with the “Explore” (based on the US Clinical Practice Guidelines) (22), the “QuitGuide” (based on the US Clinical Practice Guidelines), and the “iCanQuit” (based on acceptance and commitment therapy) (23). Other promising findings were that, compared with the initial stage, participants were more satisfied with the program at the end of the program. Also, there was a significant increase in participants who believed/agreed this program can help them to quit smoking from baseline to the end of the program. In general, the overall satisfaction for this app was relatively high, including the design of overall quitting journey and ease of usage for crucial functions, while there is still room for improvement. For example, personalization (the assignments are customized for me) and interestingness (the task is interesting and exciting) of the interventional tasks both had the lowest satisfaction of 68%, and should be further be improved. Most users considered “CBT-based tasks for quitting smoking” and “report of quitting progress” as important factors for quitting and relatively easy to use. Meanwhile, the “SOS” and “smoking record” should be tweaked further as they are considered important for smoking cessation but not easy/convenient to use by the participants. “Twitter-like forum” and “intervention notification” are relatively less important may be because they are not considered as directly helpful in quitting smoking.

### Program Efficacy

This program demonstrated a promising high self-reported quit rate. The percentage of 33-day self-reported continuous prevalence abstinence was 63.9%. Self-reported 7-day point prevalence abstinence quit date was ranged from 78 to 88%, which was much more higher than the text messaging-based “Happy Quit” program (ranged from ~10 to <30%) (12). These findings provide evidence on promising effectiveness of smartphone applications as an intervention tool for smoking cessation support. Cognitive behavioral approaches were the most promising and highly recommended interventions for quitting smoking (14). An English version of CBT-based app for smoking cessation (Quit Genius) not only demonstrated the efficacy of helping users quit smoking but also the improvement of mental health and wellbeing (24).

This study found that the self-reported 33-day continuous abstinence rate was significantly higher in participants from Shanghai City (more than 70%) than those from Changsha City (<50%). Compared with participants from Changsha City, participants from Shanghai City were older aged, higher educated, received more salary, and more nicotine dependent, although previous research indicates that light smokers are more likely to quit than heavy smokers without pharmacological interventions (25), and smartphone apps can help socioeconomically disadvantaged smokers to increase treatment exposure (26). The result of higher quit rate in participants from Shanghai City may indicate that education could plan a key role in helping people to quit smoking by app. The sociodemographic factors may have impact on abstinence rates and should be further explored in the future. With efficacy, this CBT-based smartphone app, like “CureApp Smoking Cessation” (27), can be combined with clinical standard smoking cessation treatment program to improve long-term continuous abstinence.

### Limitations

There are some limitations in this study. First, this is a small sample size, single-group cohort study that examined only the 7–14 days of pre-quit stage and 33 days of post-quit stage of app smoking cessation activities. Thus, we did not measure the long-term cessation outcomes. Second, we only recruited participants from two cities in China. Third, participants may also receive other smoking cessation interventions during the study, while none of them reported using other cessation methods. Finally, the smoking abstinence was only self-reported rather than biologically verified. It is possible that this could have biased the findings of preliminary intervention outcomes of short-term quit rates.

## Conclusions and Future Directions

Despite the limitations, the findings from this study demonstrate the feasibility and acceptability of the CBT-based smartphone app intervention for smoking cessation. Also, the significant rise in self-reported overall satisfaction with the app and the number of participants who believed/agreed this app can help them to quit smoking from baseline to the end of the program are promising. To further confirm these initial findings, a randomized controlled trial with a large sample (15) is underway to evaluate the long-term efficacy of this CBT-based app for quitting smoking among Chinese smokers. This study introduces a new digital treatment model, which has promise for overcoming barriers to accessing traditional in-person smoking cessation services and extending nationwide smoking cessation services in China.

## Data Availability Statement

All data in the current study will be available from the corresponding author on reasonable request and with completion of data user agreement.

## Ethics Statement

The studies involving human participants were reviewed and approved by the Ethics Committee of Sir Run Run Shaw Hospital, an affiliate of Zhejiang University, Medical College (No: 20200129-33). Informed consent documents were signed by all participants. The purpose, procedures and measurements, potential risks and benefits of the study were explained to each participant. The patients/participants provided their written informed consent to participate in this study.

## Author Contributions

YL conceived the study, collected the data, and took the lead in interpreting the data and writing the manuscript. JT reviewed the literature, performed the statistical analyses, drafted the report, interpreted the data, and commented on the manuscript. Both authors contributed to the article and approved the submitted version.

## Conflict of Interest

The research was supported by Johnson & Johnson Pharmaceutical Company (K-20201478). The funder was collaboratively involved in app software development, but had no role in the data interpretation, writing of the manuscript, or submitting the paper for publication.

## Publisher's Note

All claims expressed in this article are solely those of the authors and do not necessarily represent those of their affiliated organizations, or those of the publisher, the editors and the reviewers. Any product that may be evaluated in this article, or claim that may be made by its manufacturer, is not guaranteed or endorsed by the publisher.

## Abbreviations

CBT: cognitive behavioral therapy.
