# Supplementary material for: Feasibility and Acceptability of a Cognitive Behavioral Therapy-Based Smartphone App for Smoking Cessation in China: A Single-Group Cohort Study
Source: Front Psychiatry. 2022 Mar 3;12:759896. doi: 10.3389/fpsyt.2021.759896 (PMC8928122; doi:10.3389/fpsyt.2021.759896)
Supplement: Supplementary file 1 [file Data_Sheet_1.docx]

**CBT-based app user journey introduction**

**(1) Register and login**

User need to register an account in the app via mobile phone number and text message verification. Basic information input will be needed for the first time, including nickname, gender and age (**S Figure 1**).


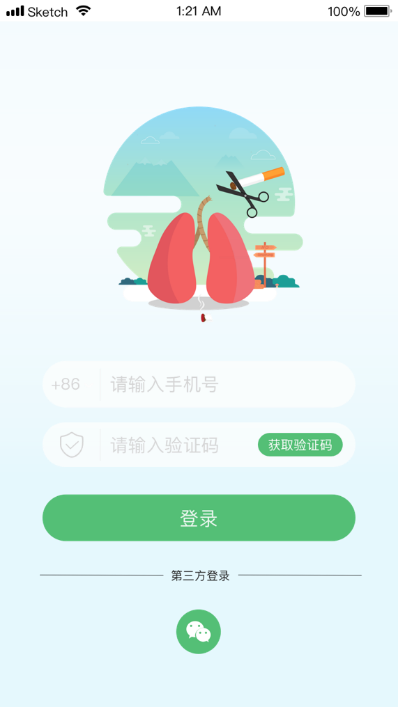

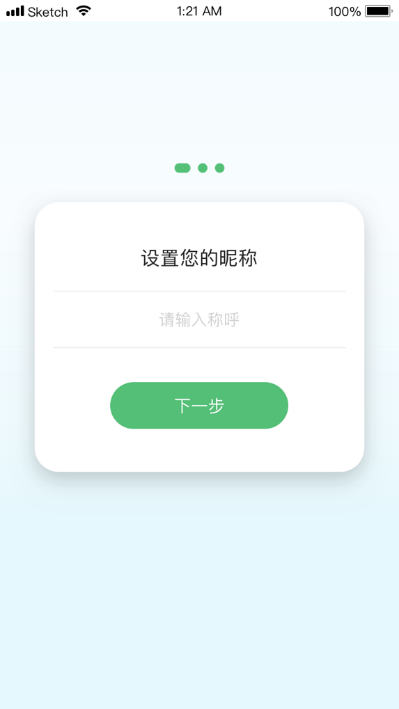


**S Figure 1** App register and login page

**(2) Preparation stage**

At this stage, user will follow app journey to complete the pr-quitting preparation (**S Figure 2**).


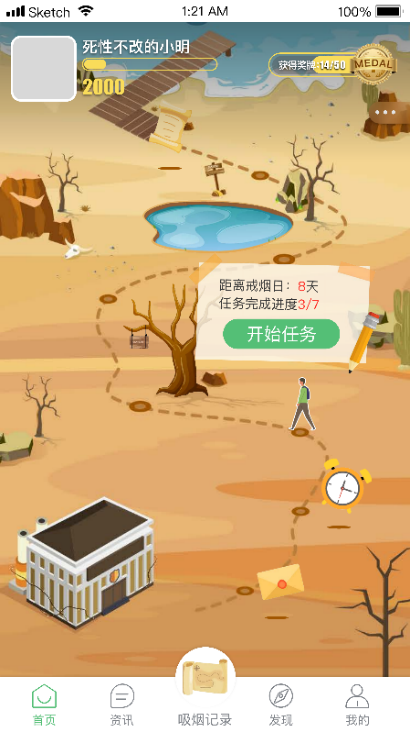


**S Figure 2** App main page of preparation stage

a. User will take a survey to get the pre-quit evaluation and personalized report including FTCD level, BMI and quitting suggestion.

b. User will set a quit date in 7-14 days and select the quitting method (quit immediately on quit date, or gradually reducing the cigarette amount before quit date)

c. User will be assigned personalized daily CBT intervention information/tasks based on profile type (FTCD level, BMI, quitting method, quitting experience), to better prepare themselves for quitting from both psychological and behavioral aspects. They cannot move to the next step before confirming if they have finished the provided task. And they will be free to view any information already unlocked. An example of CBT training task is showed in **S Figure 3**.

**
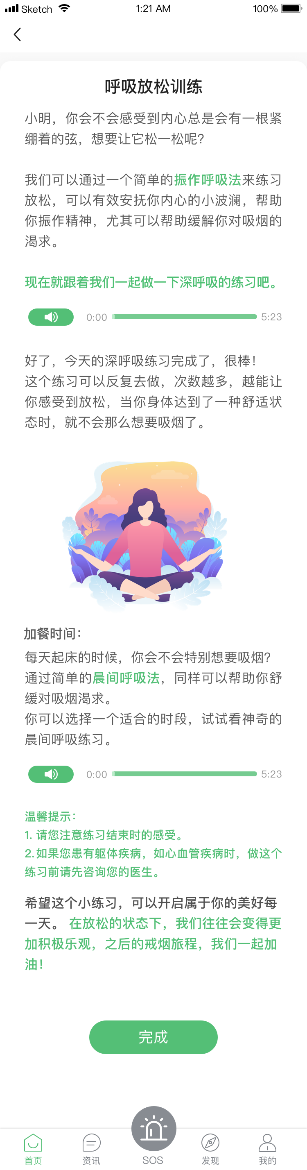
**

**S Figure 3** An example of CBT training task content- breathing relaxation training

d. User will get virtual reward in the journey map at different stages after they complete certain tasks.

e. Smoking record: user will be encouraged to monitor and record their smoking behavior. They can record time, location, craving triggers using smoking record function (**S Figure 4**) and app will provide an analysis and summary.


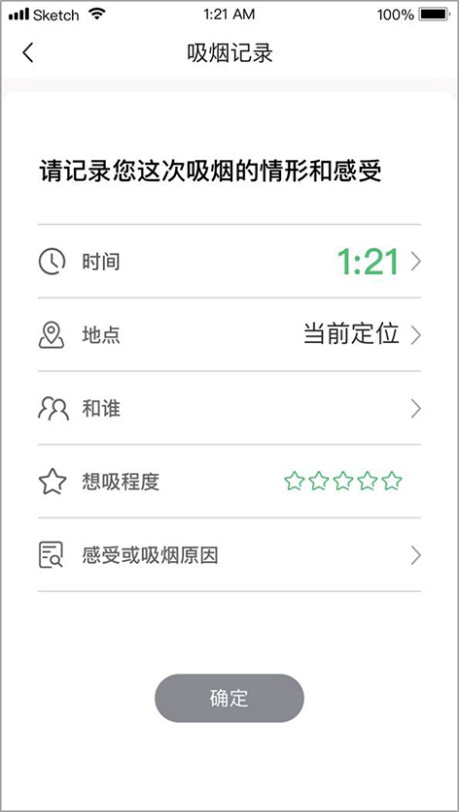


**S Figure 4** Smoking record input page

f. After the preparation task completion, user will be encouraged to make a quit manifesto on the quit date they set. Two types of method provided for creating manifesto: by video & poster (**S Figure 5**).


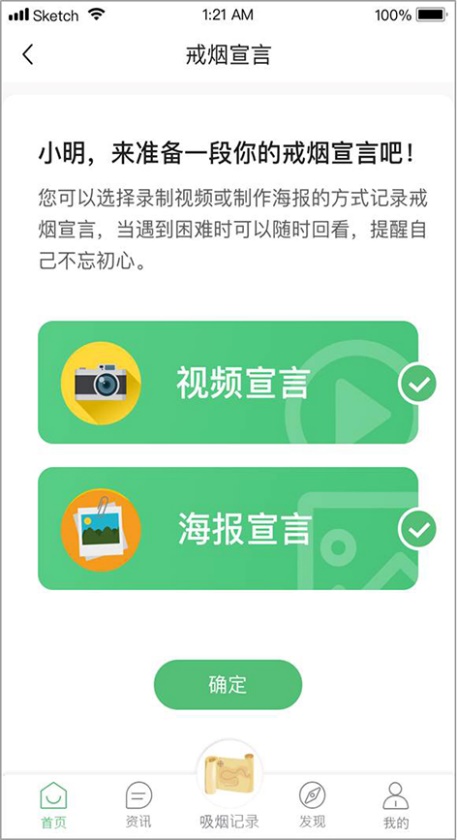


**S Figure 5** Quit manifesto page

**(3) Cessation stage**

The app journey main page will be updated to a new ocean map when user completed the quit manifesto on the quit date, and user will start the post-cessation stage journey which will be last for 30 days (**S Figure 6**).


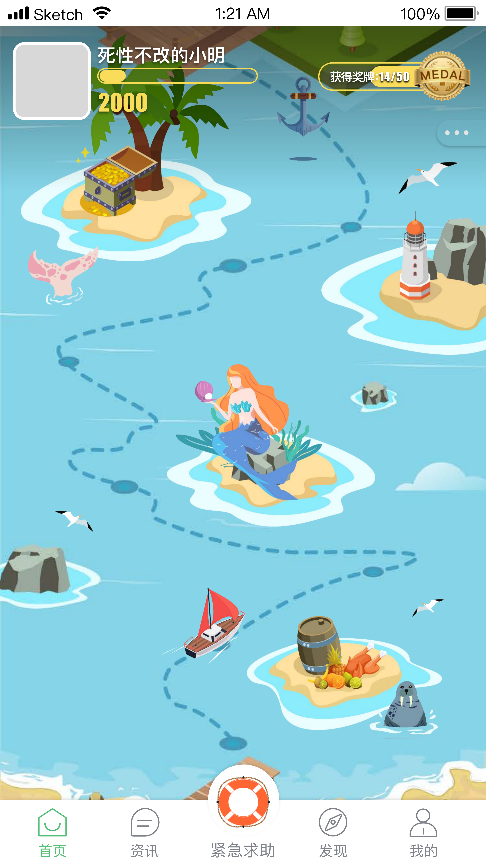


**S Figure 6** Main page of post-cessation stage

a. User will be assigned personalized daily CBT intervention information/tasks based on profile type (FTCD level, BMI, quitting method, quitting experience) to deal with the post-quitting challenges including withdraw symptoms at early cessation stage, and to enhance self-recognition for achievement. They will be free to view any information already unlocked. An example of CBT training task is showed in **S Figure 7**.


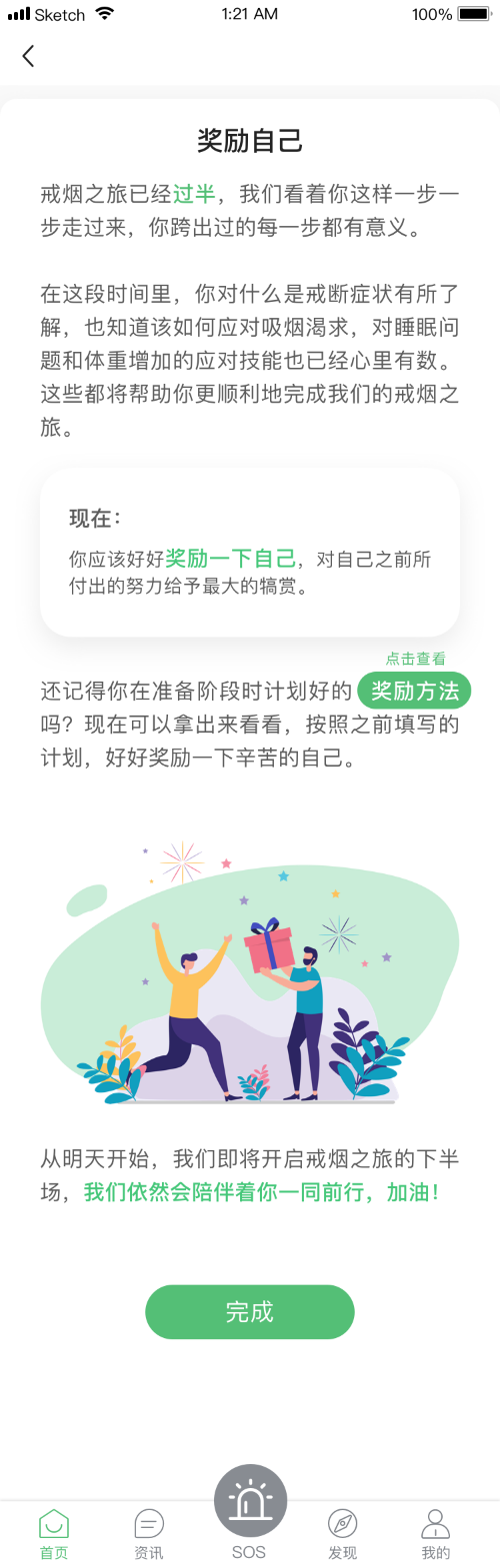


**S Figure 7** An example of CBT training task content- periodic self-reward

b. The app will send notifications at different stages of the quitting process, such as motivational messages, tips for craving based on the personalized smoking behavior reflected from smoking record for each user (**S Figure 8**).


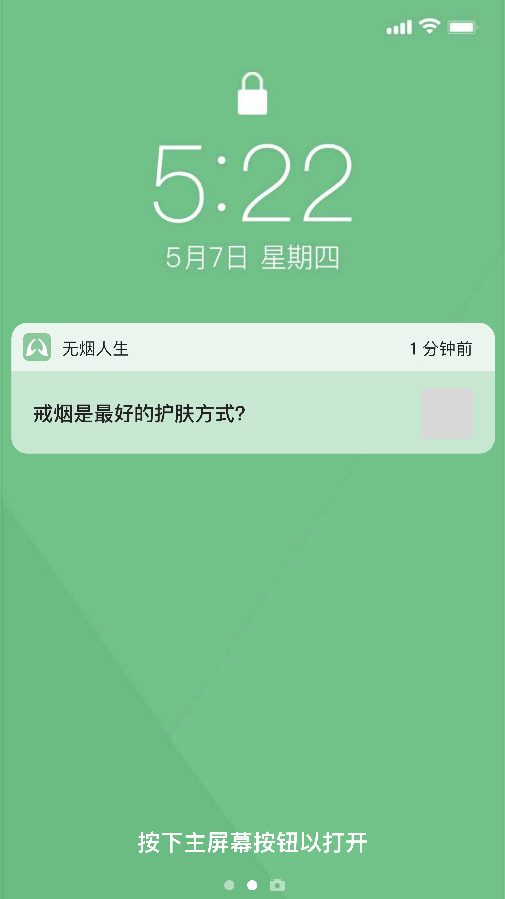


**S Figure 8** An example of notification

c. User also can seek for help SOS function (emergency help) during cessation stage. They can select the withdrawal symptoms and get corresponding solutions (**S Figure 9**).


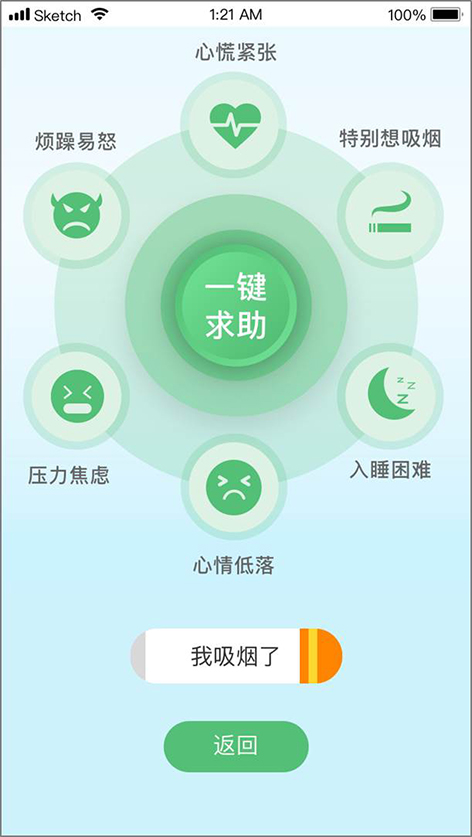


**S Figure 9** SOS function page

d. The app includes a quitting benefits tracker that will notify the user about the health benefits and financial benefits gained after quitting (**S Figure 10**)


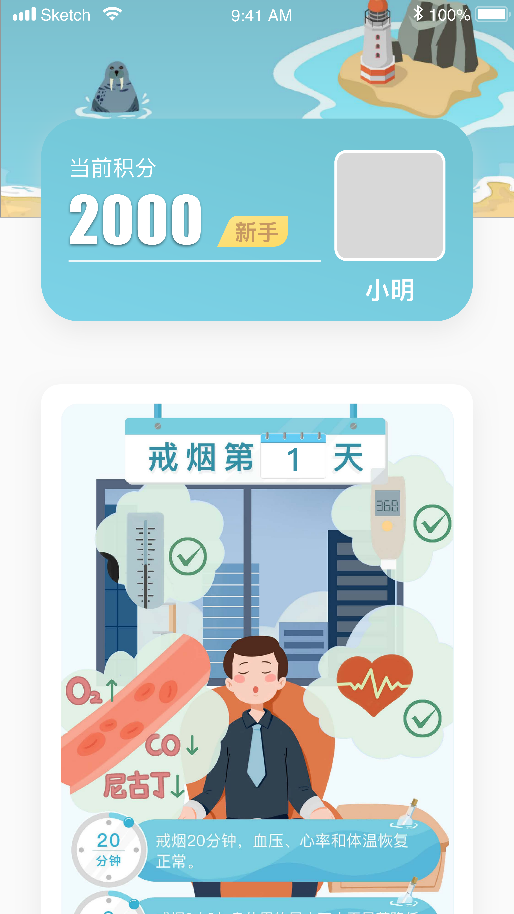

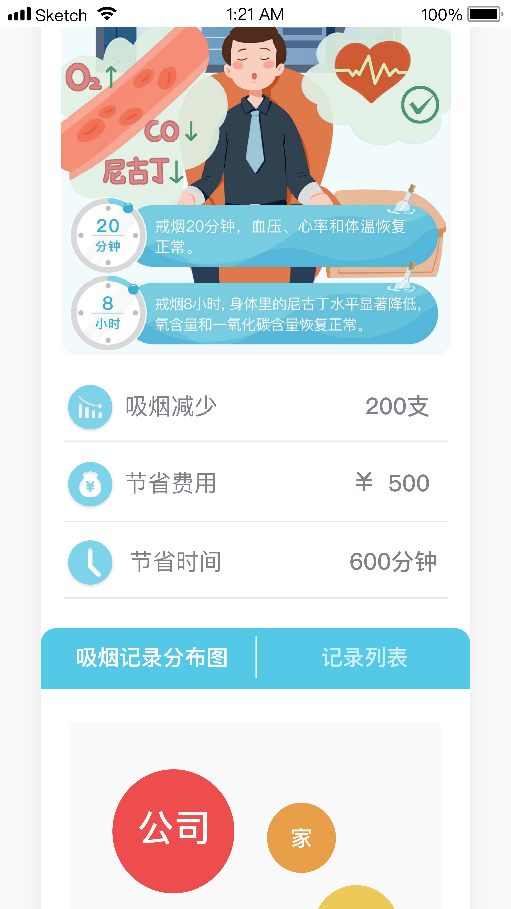


**S Figure 10** Quitting benefits tracker page

**(4) Social interaction and information function**

App includes social interaction module of a twitter-like forum for user interaction and smoking cessation information education module for user to check and learn freely (**S Figure 11**).

**
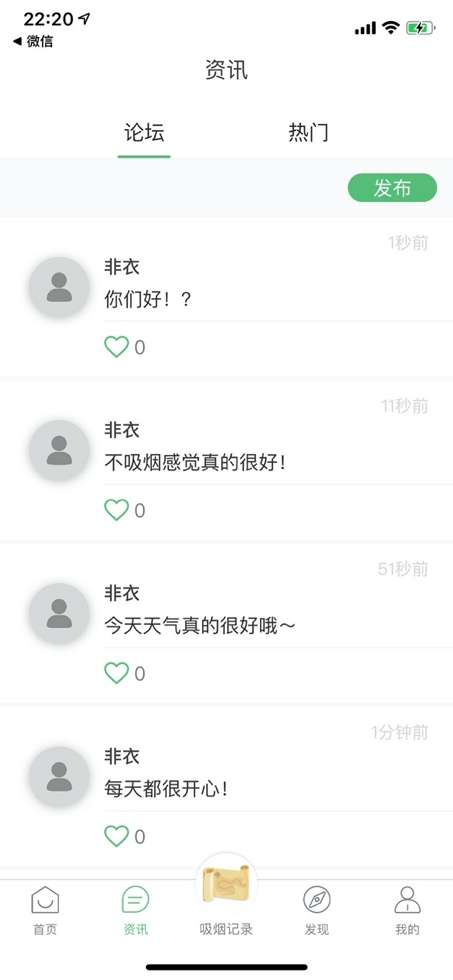

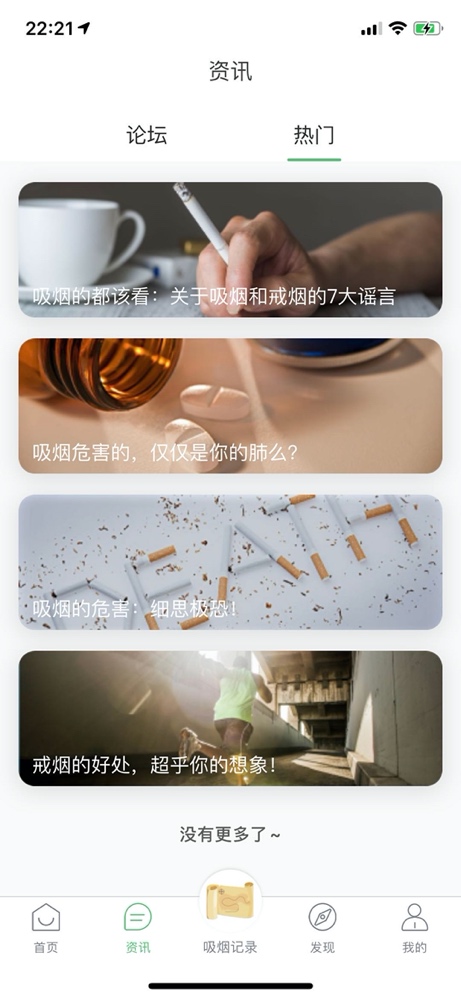
**

**S Figure 11** Twitter-like forum and information module

**(5) Login time**

Non-quitters are also more likely to log in afternoon, Shanghai users access the app more actively in the morning. While Changsha users are more active than Shanghai users in the afternoon after 4 PM (**S Figure 12**)
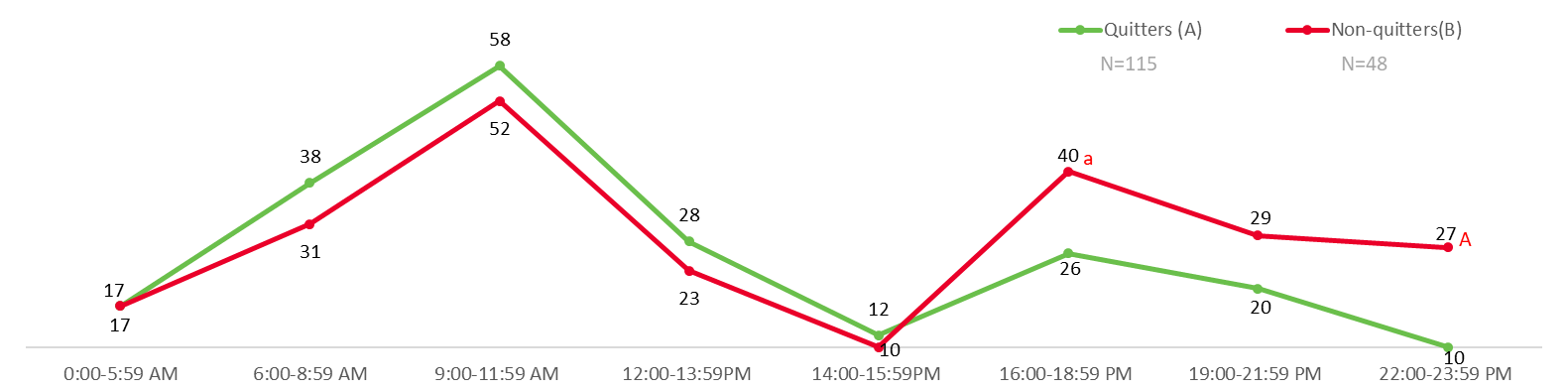


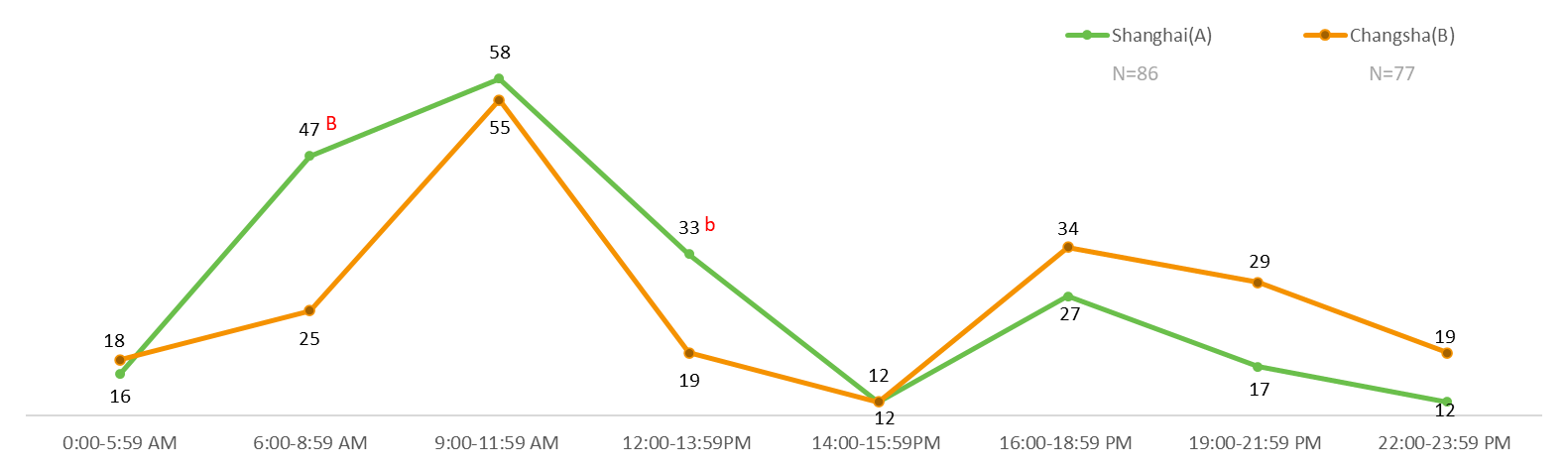


**S Figure 12** Percentage of active users (Login to the app) at different time period of day by quitters and none-quitters, and by Shanghai city and Changsha city during post-quit stage (%), (A, B: p<0.05; a, b: p<0.01)

**(6) Recommendation to friends**

Compared with initial stage, participants are more likely to recommend to friends the end of the program (**S Figure 13**).


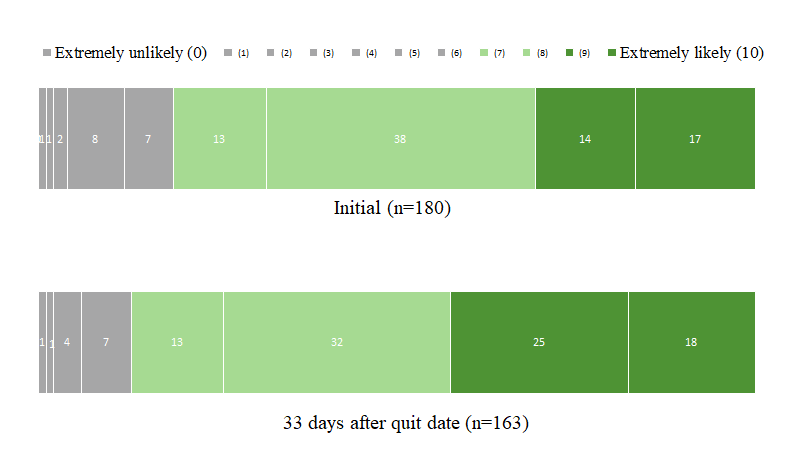


**S Figure 13** Assessment of recommendation to friends at initial stage and the end of the program (%)
