# Supplementary material for: Feasibility and Acceptability of a Cognitive Behavioral Therapy-Based Smartphone App for Smoking Cessation in China: A Single-Group Cohort Study
Source: Front Psychiatry. 2022 Mar 3;12:759896. doi: 10.3389/fpsyt.2021.759896 (PMC8928122; doi:10.3389/fpsyt.2021.759896)
Supplement: Supplementary file 2 [file Data_Sheet_2.docx]

**S Table 1** Overall Satisfaction (very satisfied and satisfied) with the CBT-based smoking cessation app across stages

by subgroup (%)

|  | City | | Age | | FTCD | | | Smoking status | | Cigarettes / day | |
| --- | --- | --- | --- | --- | --- | --- | --- | --- | --- | --- | --- |
|  | Shanghai | Changsha | <35y | 36-45y | Light | Moderate | Heavy | Quitter | Non-Quitter | <10 | >10 |
| Number of participants | 89 | 81 | 120 | 50 | 47 | 82 | 41 | 115 | 48 | 34 | 129 |
| Initial (%) | 94 | 92 | 94 | 92 | 92 | 93 | 95 | 92 | 96 | 94 | 93 |
| Preparation (%) | 92 | 91 | 93 | 90 | 88 | 91 | 98 | 94 | 90 | 88 | 94 |
| 7 days (%) | 93 | 89 | 91 | 92 | 85 | 91 | 98 | 93 | 89 | 85 | 93 |
| 15 days (%) | 97 | 82 | 89 | 92 | 83 | 93 | 93 | 93 | 83 | 82 | 92 |
| 33 days (%) | 98 | 97 | 98 | 96 | 96 | 97 | 100 | 97 | 98 | 97 | 98 |

Data are in %

FTCD: the Fagerström Test for Cigarette Dependence

Light smoking: total FTCD mean scores < 4

Moderate smoking: total FTCD mean scores 4-6

heavy smoking: total FTCD mean scores between 7 to 10

Quitters: self-reported 33-day continuous abstinence

**S Table 2** Participants who believed/agreed this app can help them to quit smoking across stages by subgroup (%)

|  | City | | Age | | FTCD | | | Smoking status | | Cigarettes / day | |
| --- | --- | --- | --- | --- | --- | --- | --- | --- | --- | --- | --- |
|  | Shanghai | Changsha | <35y | 36-45y | Light | Moderate | Heavy | Quitter | Non-Quitter | <10 | >10 |
| Number of participants | 89 | 81 | 124 | 51 | 47 | 82 | 41 | 115 | 48 | 34 | 129 |
| Initial (%) | 76 | 62 | 67 | 73 | 68 | 68 | 72 | 73 | 60 | 65 | 71 |
| Preparation (%) | 83 | 74 | 80 | 76 | 81 | 76 | 83 | 80 | 73 | 71 | 80 |
| 7 days (%) | 89 | 80 | 83 | 88 | 78 | 84 | 93 | 87 | 79 | 74 | 88 |
| 15 days (%) | 92 | 81 | 86 | 88 | 78 | 89 | 93 | 90 | 79 | 76 | 90 |
| 33 days (%) | 98 | 96 | 98 | 94 | 91 | 99 | 100 | 97 | 96 | 91 | 98 |

Data are in %

FTCD: the Fagerström Test for Cigarette Dependence

Light smoking: total FTCD mean scores < 4

Moderate smoking: total FTCD mean scores 4-6

heavy smoking: total FTCD mean scores between 7 to 10

Quitters: self-reported 33-day continuous abstinence

Heavy FTCD participants also show higher agreement on that this app is helpful on smoking cessation

**S Table 3** Demographic and smoking characteristics between quitters and none-quitters (only included 163 completed participants)

|  | Quitters (N=115) | None - quitters (N=48) | P-value |
| --- | --- | --- | --- |
| **Demographic characteristics** |  |  |  |
| Age (mean ± SD) | 31.5 ± 6.83 | 32.2 ± 5.21 | 0.532 |
| 20-30 years old (n, %) | 57 | 18 | 0.216 |
| 31-45 years old (n, %) | 58 | 30 |  |
| Gender (n, %) |  |  |  |
| Male | 99 | 45 | 0.26 |
| Female | 16 | 3 |  |
| City (n, %) |  |  |  |
| Shanghai | 71 | 15 | <0.001 |
| Changsha | 44 | 33 |  |
| Education (n, %) |  |  |  |
| High school to College | 59 | 27 | 0.69 |
| Bachelor and above | 56 | 21 |  |
| Monthly household income (n, %) |  |  |  |
| <15,000 CNY | 36 | 16 | 0.95 |
| ≥15,000 CNY | 79 | 32 |  |
| Marital status (n, %) |  |  |  |
| Unmarried | 47 | 19 | 0.378 |
| Married (without child/children) | 12 | 2 |  |
| Married (with child/children) | 56 | 27 |  |
| **Smoking characteristics** |  |  |  |
| Years of smoking (n, %) |  |  |  |
| 1-5 years | 41 | 18 | 0.964 |
| >5 years | 74 | 30 |  |
| Smoked cigarettes per day (n, %) |  |  |  |
| 5-10 cigarettes | 57 | 18 | 0.216 |
| >10 cigarettes | 58 | 30 |  |
| FTCD mean score (mean ± SD) | 4.8 ± 1.98 | 4.6 ± 2.18 | 0.604 |
| Light to moderate smoking (n, %) | 54 | 25 | 0.671 |
| Heavy smoking (n, %) | 61 | 23 |  |
| Quit attempts during the past 12 months (n, %) |  |  |  |
| No | 0 | 0 | 1 |
| Yes | 115 | 48 |  |
| Selection of quitting smoking way^#^ (n, %) |  |  |  |
| Gradually quit | 77 | 34 | 0.764 |
| Abruptly quit | 38 | 14 |  |
| Days of preparation stage^#^ (n, %) |  |  |  |
| 7 days | 61 | 32 | 0.153 |
| 8-14 days | 54 | 16 |  |

FTCD: the Fagerström Test for Cigarette Dependence; Light to moderate smoking: total FTCD mean scores ≤ 6; heavy smoking: total FTCD mean scores between 7 to 10.

Quitters: self-reported 33-day continuous abstinence

**S Table 4** Average login times (number of times opened the app) across all stages

| Stage | Frequency (times/day) % | | | | Mean ± SD | Median |
| --- | --- | --- | --- | --- | --- | --- |
|  | ≤1 | 2 | 3 | >3 |  |  |
| Average login times in pre-quit stage | 11 | 33 | 29 | 27 | 2.75 ± 2.04 | 2.14 |
| Average login times in overall post-quit stage | 15 | 50 | 26 | 8 | 1.77 ± 0.93 | 1.53 |
| Average login times for pre-quit stage + post-quit 15 days | 12 | 44 | 25 | 19 | 2.13 ± 1.20 | 1.77 |
| Average login times in post-quit 1-7 days | 18 | 41 | 25 | 16 | 1.99 ± 1.17 | 1.86 |
| Average login times in post-quit 8-15 days | 24 | 52 | 16 | 9 | 1.65 ± 0.98 | 1.50 |
| Average login times in post-quit 16-33 days | 26 | 45 | 20 | 10 | 1.68 ± 0.96 | 1.47 |
